# Researches on the Blood

**Published:** 1843-06

**Authors:** 


*Researches on the Blood.*—A late treatise by Andral, Gavaret, and Delafond, on the condition of the blood in animals in health and in disease, contains the following statements: The quantity of fibrin normally present in the blood differs greatly in different animals, and holds no strict relation to energy of constitution. The quantity of fibrin is very small in the blood of animals examined within a day after their birth. During the latter period of gestation the fibrin of the blood diminishes to below its normal amount; but immediately after parturition and during the early period of lactation the blood contains its largest proportion of fibrin, and puerperal diseases are apt to occur correlatively with its amount. The blood-globules exist proportionally in greater quantity in the blood of carnivorous, and in least quantity in that of herbivorous animals. The greater proportion of globules in the blood is correlative with greater constitutional vigour in individuals of the same species; and improved breeds of sheep are distinguished by an increase in the number of these globules. During gestation, after parturition, and within a day after birth, the globules are diminished or increased correlatively with the fibrin. The serum of the blood is in smallest proportional amount in carnivorous animals, and most abundant in the herbivora. It accrues considerably during the occurrence of pot-belly (*cachexia aqueuse*) in sheep, conjointly with a notable decrease of the fibrin and globules. Dropsy only supervenes as a consequence of an alteration in the blood, when this fluid is divested of a certain quantity of its albumen. The diminution of the blood-globules alone is not sufficient to produce it; hence dropsy is not attendant on chlorosis. But it exists in man when albuminous urine is excreted, and in sheep when the parasite *fasciola hepatica* is found in the liver, a circumstance which is often attended by pot-belly and anemia.—*Lond. Lancet*, from *Gaz. des Hop.*

